# Analysis of polycyclic aromatic hydrocarbons (PAHs) and their derivatives in biochar treated stormwater

**DOI:** 10.1016/j.mex.2023.102232

**Published:** 2023-05-30

**Authors:** Alberto Celma, Anna-Karin Dahlberg, Karin Wiberg

**Affiliations:** Department of Aquatic Sciences and Assessment, Swedish University of Agricultural Sciences (SLU), SE-750 07, Uppsala, Sweden

**Keywords:** Polycyclic aromatic hydrocarbons (PAHs), Oxygenated polycyclic aromatic hydrocarbons (oxy-PAHs), Nitrogen heterocyclic polycyclic aromatic compounds (N-PACs), Biochar, Surface water, Sample preparation, *Polycyclic aromatic compounds extraction*

## Abstract

Polycyclic aromatic hydrocarbons (PAHs), oxygenated PAHs (oxy-PAHs) and nitrogen heterocyclic polycyclic aromatic compounds (N-PACs) are persistent and semi-volatile organic compounds primarily formed due to incomplete combustion of organic material or, in the case of the derivatives, through transformation reactions of PAHs. Their presence in the environment is ubiquitous and many of them have been proven carcinogenic, teratogenic, and mutagenic. These toxic pollutants can therefore pose a threat to both ecosystem and human health and urges for remediation strategies for PAHs and derivatives from water bodies. Biochar is a carbon-rich material resulting from the pyrolysis of biomass resulting in a very porous matter with high surface area for an enhanced interaction with chemicals. This makes biochar a promising alternative for filtering micropollutants from contaminated aquatic bodies. In this work, a previously developed and validated methodology for the analysis of PAHs, oxy-PAHs and N-PACs in surface water samples was adapted for its utilization in biochar treated stormwater with special emphasis on scaling down the solid-phase extraction as well as including an additional filtering step for the removal of particulate matter in the media.

• Optimized extraction method for PAHs, oxy-PAHs and N-PACs from stormwater treated with biochar.
• Biochar strongly impacts the stormwater matrix and, therefore, additional steps are required in the extraction methodology.
• Solid-phase extraction combined with GC–MS have been used to analyse PAHs, oxy-PAHs and N-PACs in stormwater treated with biochar.

## Graphical abstract

**Figure:**
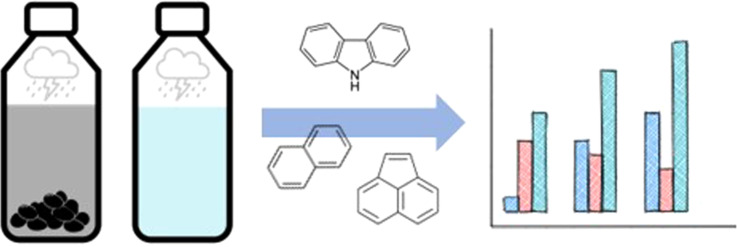

Specifications table

**Table:** 

Subject area:	Environmental Science
More specific subject area:	*Stormwater analysis*
Name of your method:	*Polycyclic aromatic compounds extraction*
Name and reference of original method:	*Nguyen, M.A., Ahrens, L., Gustavsson, J., Josefsson, S., Laudon, H., Wiberg, K., 2018. The Role of Spring Flood and Landscape Type in the Terrestrial Export of Polycyclic Aromatic Compounds to Streamwater. Environ. Sci. Technol. 52, 6217–6225.*https://doi.org/10.1021/acs.est.7b04874
Resource availability:	*Stormwater, solid-phase extraction, gas chromatography-mass spectrometry.*

## Introduction

Polycyclic aromatic hydrocarbons (PAHs) are persistent and semi-volatile organic compounds primarily formed due to incomplete combustion of organic material, *e.g.* petroleum, oil, gas or coal [17]. These chemicals share the peculiarity of having at least two fused aromatic rings in their chemical structure which provides them with a pronounced hydrophobic and lipophilic character [1]. Their presence in the environment in ubiquitous because of several natural and anthropogenic sources. Additionally, several other polycyclic aromatic compounds (PACs), such as oxygenated polycyclic aromatic hydrocarbons (oxy-PAHs) and nitrogen heterocyclic PACs (N-PACs) are formed during the same kind of processes or through transformation reactions with PAHs [11]. PAHs and their derivatives are widely detected in the environment, especially at sites impacted by activities that use (or used) fossil fuels or products made from fossil fuels, such as gasworks, fuel refining, heavily trafficked roads, production and use of of coke, coal tar and creosote. PAHs are also often found in aquatic bodies such as lakes, rivers, aquifers and even drinking water [1]. Their ubiquitous presence in the environment can pose a threat to both ecosystem and human health since many of these compounds have been proven carcinogenic, teratogenic and mutagenic [4,9,13,18].

Due to the hydrophobic nature of PACs, their occurrence in aquatic samples is often at lower concentrations than in soils and sediments [1]. However, PACs enter the aquatic environment via atmospheric particulate deposition, run-off from contaminated areas, leakage from contaminated soils, urban runoff, and industrial and wastewater effluents [1]. Removal of PAHs and derivatives in urban stormwater before being released to the environment can be an efficient approach to reduce the environmental exposure of traffic derived PACs [5,20].

Biochar is a carbon-rich material resulting from the pyrolysis of biomass or organic waste, such as woodchips or wastewater sludge [3,7]. Such material is mainly composed of amorphous carbon and interspersed voids [7] resulting in a very porous matter with high surface area with high capacity for interaction with chemical substances in aqueous solutions [3] making biochar a promising alternative for filtering micropollutants from contaminated water bodies. There are several studies investigating the use of biochar-enriched media as a filter for organic micropollutants in the aquatic environment [2,6,8,16], with some of them focusing on stormwater filtering [3,10,15,19].

In this work, a previously developed and validated methodology for the analysis of PAHs, oxy-PAHs and N-PACs in surface water samples [14] has been adapted for its utilization for biochar treated stormwater. As part of a project for the evaluation of biochar as a tool for filtering PAHs and derivatives from stormwater streams, a method for extracting and analysing the biochar treated stormwater, which contained biochar residues was needed. Special efforts were devoted to scaling down the solid phase extraction and the inclusion of additional filtering steps to remove particulate matter from the aqueous phase.

## Method

### Materials and methods

#### Chemicals and materials

The chemicals assessed in this study comprised a group of 17 PAHs, 10 oxy-PAHs and 4N-PACs. The PAHs analysed were: acenaphthene, acenaphthylene, anthracene, benz[a]anthracene, benzo[a]pyrene, benzo[b]fluoranthene, benzo[g,h,i]perylene, benzo[k]fluoranthene, chrysene, dibenzo[a,h]anthracene, fluoranthene, fluorene, indeno[1,2,3-c,d]pyrene, naphthalene, perylene, phenanthrene and pyrene. The oxy-PAHs analysed were: 1-indanone, 2-methylanthracene-9,10-dione, 4H-cyclopenta[def]phenanthrenone, 6H-benzo[cd]pyren-6-one, 7H-benz[de]anthracen-7-one, 9-fluorenone, anthracene-9,10-dione, benzo[a]anthracene-7,12-dione, benzo[a]fluorenone and naphthacene-5,12-dione. Finally, the N-PACs were: acridine, benzo[h]quinoline, carbazole and quinoline. The isotopically labelled internal standards (ILIS) used were: [^2^H_8_]-9-fluorenone, [^2^H_10_]-acenaphthene, [^2^H_8_]-acenaphthylene, [^2^H_10_]-anthracene, [^2^H_8_]-anthracene-9,10-dione, [^2^H_12_]-benz[a]anthracene, [^2^H_12_]-benzo[a]pyrene, [^2^H_12_]-benzo[b]fluoranthene, [^2^H_12_]-benzo[g,h,i]perylene, [^2^H_12_]-benzo[k]fluoranthene, [^2^H_8_]-carbazole, [^2^H_12_]-chrysene, [^2^H_14_]-dibenzo[a,h]anthracene, [^2^H_10_]-fluoranthene, [^2^H_10_]-fluorene, [^2^H_12_]-indeno[1,2,3-c,d]pyrene, [^2^H_8_]-naphthalene, [^2^H_12_]-perylene, [^2^H_10_]-phenanthrene, [^2^H_10_]-pyrene and [^2^H_7_]-quinoline. All reference compounds were purchased from Sigma-Aldrich, CDN isotopes and Cambridge Isotope Laboratories with purities ranging from 95 to 100%. The recovery standards (InjS) were [^13^C]-PCB-97 and [^13^C]-PCB-188 (Cambridge Isotope Laboratory, UK).

Glass microfibers filters (GF/C, 47 mm, 1.2 µm pore size) were purchased in Whatman (UK). Oasis hydrophilic−lipophilic balance (HLB) cartridges (6 mL, 200 mg, 30 µm) were purchased from Waters (Milford, MA, USA). Dichloromethane suprasolv-grade (DCM), methanol hypergrade for LC-MS-grade (MeOH), toluene suprasolv-grade and sodium sulphate anhydrous for analysis grade were purchased at Supelco (Sigma-Aldrich). Millipore water was produced in-house by filtration through a MilliPak 0.22 µm filter.

Calibration standards were prepared from stock solutions of PAHs and derivatives in pure toluene and ranged from 1 to 1000 ng mL^−1^ (eight calibration points), with ILIS at 100 ng mL^−1^ in both calibration standards and samples.

#### Extraction method

The proposed extraction methodology has been adapted from previously validated analytical methodology for the large-volume extraction of PAHs and derivatives in surface water [14]. However, the current methodology is now optimized for smaller volumes of biochar treated stormwater.

**Fig. 1:**
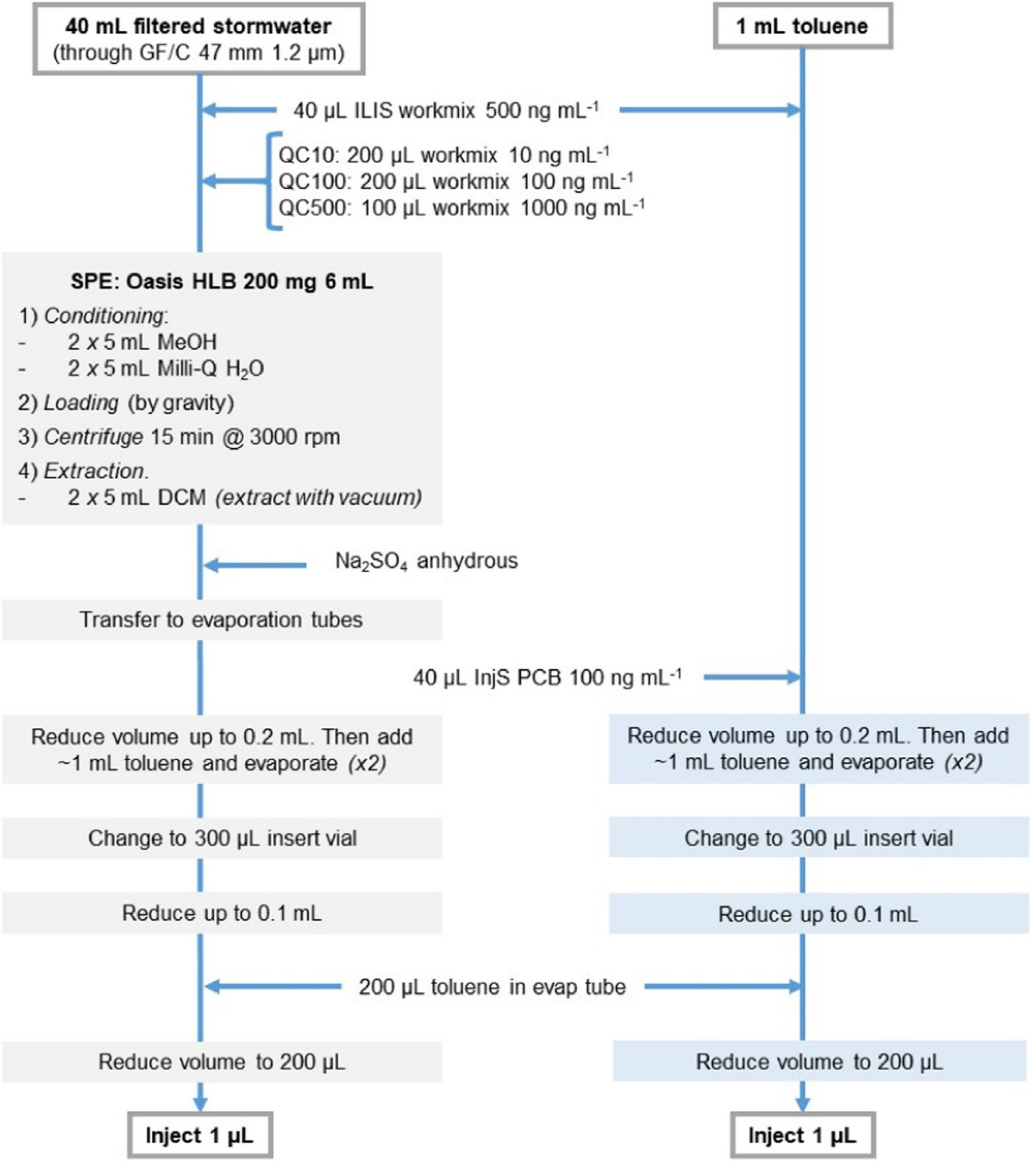
Schematic workflow for extraction of PAHs and derivatives in stormwater treated with biochar. On the left, extraction procedure for stormwater samples; on the right, recovery standards extraction. (*DCM*: dichloromethane; *ILIS*: isotopically labelled internal standards; *InjS*: recovery standards; *MeOH*: methanol; *QC*: quality control).

To that purpose, stormwater treated with biochar was filtered through glass microfiber disk filters (Whatman GF/C 47 mm) under vacuum to remove the suspended solid particles originating from the biochar contamination. After filtration, an aliquot of 40 mL was separated and spiked with a mixture of ILIS to act as surrogate for PAHs and derivatives. Samples were extracted by means of Oasis HLB (200 mg, 6 mL, Waters Corporation, UK) SPE cartridges, with sample being loaded by gravity. After drying SPE cartridges under a gentle back pressure, they were centrifuged at 3000 rpm for 15 min (Eppendorf centrifuge 5810, Germany) to remove the remaining water content in the stationary phase. Extraction was done with 2 × 5 mL of DCM, and the remaining moisture in the organic solvent was dried out with the addition of anhydrous Na_2_SO_4_. The organic phase was then transferred to evaporation tubes and the volume reduced to 200 µL under a gentle stream of N_2_ (N-EVAP nitrogen evaporator, Organomation Associates inc., MA, US). After that, approximately 1 mL of toluene was added and evaporated until 200 µL twice to ensure no traces of DCM were left in the extract. Extract was then transferred to a vial and 200 µL of toluene was used to clean the evaporation tube and added to the vial. Extract volume was then adjusted to a final volume of 200 µL. Finally, 1 µL of extract was injected in the GC–MS system. A complete workflow of the procedure can be found in Fig. 1.

#### Instrumentation

Instrumental analysis for PAHs and derivatives was adapted from Lundstedt et al. [12]. In brief, 1 µL of extract was injected in pulsed splitless mode into an Agilent 7890B GC coupled to an Agilent 5977A MSD mass spectrometer. The injector temperature was kept at 290 °C with a pressure of 32 psi. Separation took place in a DB-5MS capillary column (60 m x 0.25 mm i.d.) with Helium at 2 mL min^−1^ as the carrier gas. A programmed temperature gradient was applied as follows. First, the oven was kept at 70 °C for 2 min, then the temperature increased at a rate of 30 °C min^−1^ to 125 °C. After that, the temperature increased at 5 °C min^−1^ to 310 °C, where it was kept for 5 min. The chromatographic total run time was 56 min. Auxiliary gas was kept at 310 °C. Detection was performed in a single quadrupole MS system equipped with an electron impact (EI) ionization source at 300 °C. Data acquisition was done in selected ion mode (SIM).

Data on chromatographic retention time (RT), quantifier ions as well as the corresponding ILIS used can be found in Table 1.

**Table 1:** Retention time (RT, min) and quantifier ions (*m/z*) for the targeted PAHs, oxy-PAHs and N-PACs and corresponding isotopically labelled internal standard (ILIS).

Native compound			Isotopically labelled internal standard (ILIS)		
Compound	RT (min)	*m/z*	Compound	RT (min)	*m/z*
Naphthalene	8.57	128.0	[^2^H_8_]-naphthalene	8.53	136.0
Quinoline	9.36	129.0	[^2^H_7_]-quinoline	9.33	136.0
1-Indanone	10.02	132.0	[^2^H_7_]-quinoline	9.33	136.0
Acenaphthylene	13.33	152.1	[^2^H_8_]-acenaphthylene	13.28	160.1
Acenaphthene	14.05	154.1	[^2^H_10_]-acenaphthene	13.90	164.1
Fluorene	16.03	166.1	[^2^H_10_]-fluorene	15.93	176.1
9-Fluorenone	19.23	180.0	[^2^H_8_]-9-fluorenone	19.15	188.0
Phenanthrene	20.12	178.1	[^2^H_10_]-phenanthrene	20.05	188.1
Benzo[h]quinoline	20.31	179.0	[^2^H_8_]-9-fluorenone	19.15	188.0
Anthracene	20.32	178.1	[^2^H_10_]-anthracene	20.26	188.1
Acridine	20.54	179.0	[^2^H_8_]-9-fluorenone	19.15	188.0
Carbazole	21.12	167.0	[^2^H_8_]-carbazole	20.85	175.0
Anthracene-9,10-dione	23.83	208.0	[^2^H_8_]-anthracene-9,10-dione	23.75	216.0
4H-cyclopenta[def]phenanthrenone	25.30	204.0	[^2^H_8_]-anthracene-9,10-dione	23.75	216.0
Fluoranthene	25.60	202.1	[^2^H_10_]-fluoranthene	25.50	212.1
2-methylanthracene-9,10-dione	26.35	222.0	[^2^H_8_]-anthracene-9,10-dione	23.75	216.0
Pyrene	26.60	202.1	[^2^H_10_]-pyrene	26.52	212.1
Benzo[a]fluorenone	30.60	230.0	[^2^H_8_]-anthracene-9,10-dione	23.75	216.0
Benz[a]anthracene	32.28	228.1	[^2^H_12_]-benz[a]anthracene	32.19	240.2
Chrysene	32.46	228.1	[^2^H_12_]-chrysene	32.35	240.2
7H-Benz[de]anthracene-7-one	33.00	230.0	[^2^H_8_]-anthracene-9,10-dione	23.75	216.0
Benz[a]anthracene-7,12-dione	34.46	258.0	[^2^H_8_]-anthracene-9,10-dione	23.75	216.0
Naphthacene-5,12-dione	35.67	258.0	[^2^H_8_]-anthracene-9,10-dione	23.75	216.0
Benzo[b]fluoranthene	37.07	252.1	[^2^H_12_]-benzo[b]fluoranthene	36.97	264.2
Benzo[k]fluoranthene	37.16	252.1	[^2^H_12_]-benzo[k]fluoranthene	37.08	264.2
6H-Benzo[cd]pyren-6-one	38.30	254.0	[^2^H_8_]-anthracene-9,10-dione	23.75	216.0
Benzo[a]pyrene	38.35	252.1	[^2^H_12_]-benzo[a]pyrene	38.25	264.2
Perylene	38.66	252.1	[^2^H_12_]-perylene	38.58	264.2
Indeno[1,2,3-c,d]pyrene	42.63	276.1	[^2^H_12_]-indeno[1,2,3-c,d]pyrene	42.52	288.2
Dibenzo[a,h]anthracene	42.75	278.1	[^2^H_14_]-dibenzo[a,h]anthracene	42.39	292.1
Benzo[g,h,i]perylene	43.65	276.1	[^2^H_12_]-benzo[g,h,i]perylene	43.55	288.2

### Method discussion

The methodology previously developed and validated by Nguyen et al. [14] aimed at quantifying low-trace levels of PAHs and derivatives in surface waters. To that purpose, large-volume SPE was selected as the best analytical procedure. However, the definite goal of the current methodology was to improve the throughput of samples at higher concentration levels. Hence, it was decided to scale down the SPE into a more efficient and high-throughput scale, which did not involve changes or alterations that required an additional method validation for the new procedure.

From another perspective, sample type loaded into the SPE changed from raw surface water to stormwater treated with biochar. In this sense, this was a major change that required adjustments and extra efforts to arrive at an efficient and optimized methodology. These changes are in-depth discussed in the following sections.

#### Scaling down the solid phase extraction

Instead of 12 L of raw sample extraction, the sample volume was scaled down to 40 mL, i.e. a reduction in sample size by a factor of 300. The size of the SPE cartridge was also reduced, from 6 g HLB sorbent to 200 mg (reduction factor of 30). Additionally, the conditioning and extraction solvents were also adapted to those recommended for 200 mg cartridges (Fig. 1). It should be noted at this point that the reduction factor for both SPE cartridges and solvents was far below the reduction factor for the sample. Thus, the viability of the methodology to achieve comparable performance with previously validated methodology was not compromised. Thus, it did not require an extensive method validation. Besides, quality control (QC) samples consisting of spiked samples at different concentrations levels (10, 100 and 500 ng mL^−1^) were included in all extraction batches to ensure the performance of the methodology. Additionally, injection recovery standards (InjS) were analysed alongside the samples (Fig. 1) to be able to assess losses and deviations occurring during the sample preparation process. Extraction performance was re-evaluated for the adapted methodology by the study of spiked Milli-Q samples. Such analysis showed appropriate recoveries ranging from 63 to 144% recovery. However, *6H-benzo(cd)pyren-6-one* and *benz[a]anthracene* did not show appropriate recoveries; these compounds were therefore excluded from the extraction methodology. Method detection limits (MDLs) and method quantification limits (MQLs) were in range of those previously proposed by Nguyen et al. [14].

#### The need for an extra filtration step in biochar contaminated stormwater samples

The biochar contamination strongly impacted the stormwater matrix. During method development, we observed that the powdery and hydrophobic nature of biochar [7] favoured the release of a large amounts of small non-polar particles that remained suspended in the water and thereby altered the stormwater matrix. Therefore, it was assumed that suspended particles originating from the biochar were responsible for the instant adsorption and low recoveries of ILIS. Hence, an extra filtration step was deemed necessary to remove the biochar suspended particles from the aqueous phase prior to sample extraction.

Different approaches were tested to remove biochar suspended particles with the goal of including as few steps as possible in the sample treatment with the least impact on method efficiency. Decantation, centrifugation, glass-wool column filtration and glass microfibers vacuum-aided filtration were all tested as separate treatment steps. Decantation and centrifugation were tested due to their non-invasive and limited sample manipulation required, while glass-wool and glass microfibers filters were tested as suspended particle filter materials with the expectation of limited impact on analyte loss.

**Fig. 2:**
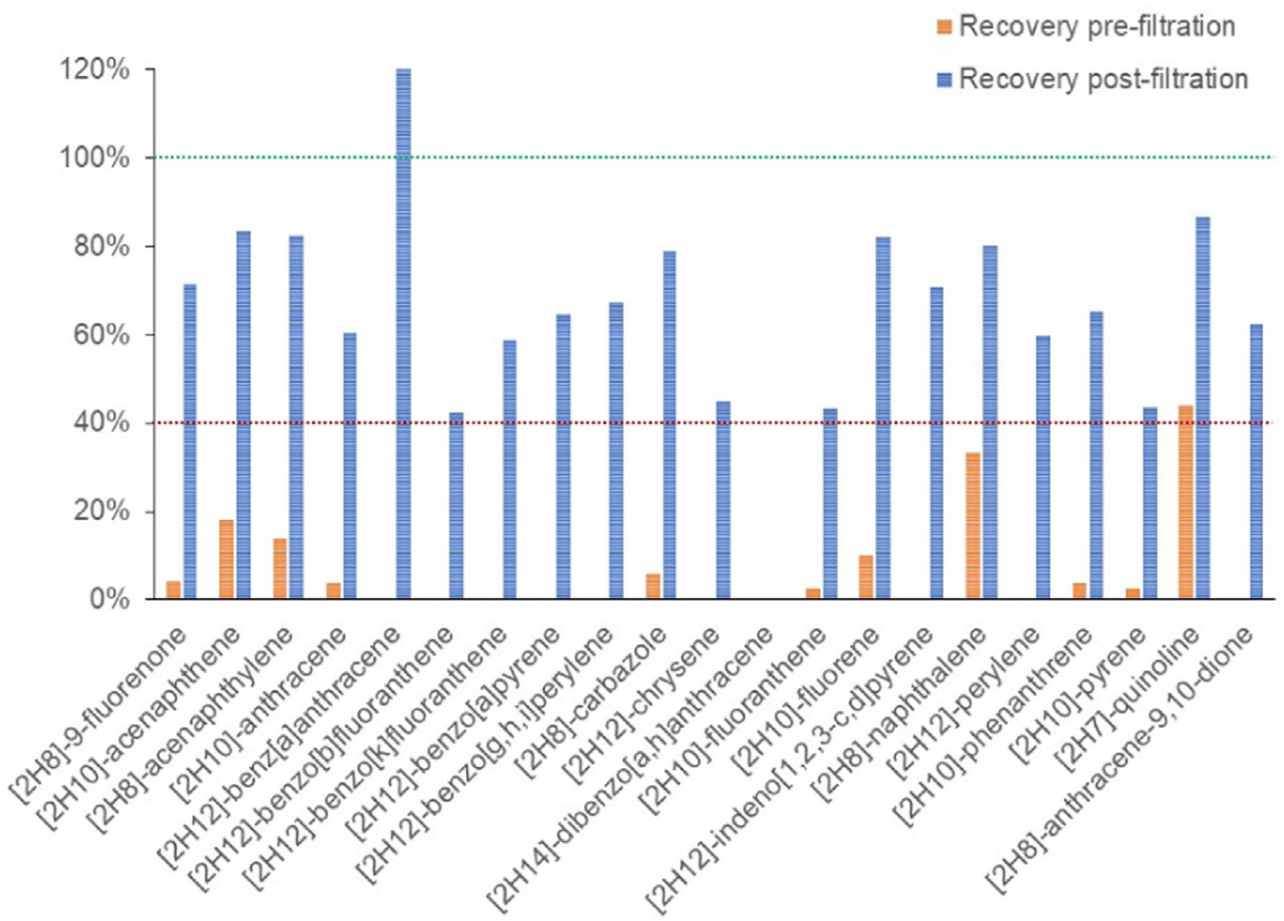
Recoveries of ILIS in pre- (orange) and post-filtration (blue) spiking experiments of stormwater treated with biochar. Green line indicates 100% recovery and red line indicates 40% recovery. Data is shown as a percentage of recovery compared to that on unfiltered spiked Milli-Q water. (For interpretation of the references to color in this figure legend, the reader is referred to the web version of this article.)

From visual inspection, it was concluded that the first two strategies, decantation and centrifugation, performed poorly on removing suspended particles due to the high hydrophobicity of biochar particles. The glass-wool column filtration helped at removing part of the biochar particles (large particles stacked at glass-wool, top layer), but only the fraction of larger suspended particles, as the filtered water still showed presence of suspended material. However, glass microfibers disk filters, with a pore size of 1.2 µm, performed well, seemingly removing suspended particles to a satisfying degree. In order to evaluate the removal efficiency of biochar particles hindering extraction of PACs by instantly adsorbing the test compounds, the recovery of ILIS was tested with fortification pre- and post-filtration, respectively. These recoveries were normalized to the recovery of ILIS when extracting spiked Milli-Q water. In the case of spiking pre-filtration, it can clearly be observed that ILIS could not be efficiently recovered from the stormwater, with some cases showing even zero recovery (Fig. 2). However, the recoveries were substantially improved when ILIS were spiked post-filtration. It should be noted at this point that these results do not indicate sorption in the glass filter. On the contrary, it explains the impact of having biochar suspended particles present in the stormwater pre filtering. Although the filtration seems to not affect the performance of the extraction of almost all analytes investigated, it did compromise the extraction of *[^2^H_14_]dibenzo[a,h]anthracene*. Thus, *dibenzo[a,h]anthracene* was excluded from the methodology.

#### Method summary

The methodology for extracting PAHs and derivatives has been adapted for the analysis of stormwater treated with biochar containing suspended particles originating from the biochar. In brief, the adaption consisted on the scaling down the SPE as well as the inclusion of an additional filtration step to remove biochar suspended particles. It should be mentioned that the addition of an extra filtration step did not significantly impact financial, timing, or environmental costs. Additionally, it was deemed necessary to exclude three substances (see Sections ``Scaling down the solid phase extraction'' and ``The need for an extra filtration step in biochar contaminated stormwater samples'') from the methodology. Thus, the new extraction method covers 15 PAHs, 9 oxy-PAHs and 4N-PACs.

## CRediT authorship contribution statement

**Alberto Celma:** Conceptualization, Methodology, Validation, Formal analysis, Investigation, Data curation, Writing – original draft. **Anna-Karin Dahlberg:** Conceptualization, Methodology, Writing – review & editing, Funding acquisition. **Karin Wiberg:** Conceptualization, Methodology, Writing – review & editing, Funding acquisition.

## Declaration of Competing Interest

The authors declare that they have no known competing financial interests or personal relationships that could have appeared to influence the work reported in this paper.

## Data Availability

Data will be made available on request.
